# Chronic lymphocytic leukemia treatment algorithm 2022

**DOI:** 10.1038/s41408-022-00756-9

**Published:** 2022-11-29

**Authors:** Paul J. Hampel, Sameer A. Parikh

**Affiliations:** Division of Hematology, Mayo Clinic, Rochester, MN USA

**Keywords:** Chronic lymphocytic leukaemia, Disease-free survival

## Abstract

The treatment landscape for patients with chronic lymphocytic leukemia (CLL) has changed considerably with the introduction of very effective oral targeted therapies (such as Bruton tyrosine kinase inhibitors and venetoclax) and next-generation anti-CD20 monoclonal antibodies (such as obinutuzumab). These agents lead to improved outcomes in patients with CLL, even among those with high-risk features, such as del17p13 or *TP53* mutation and unmutated immunoglobulin heavy chain (*IGHV*) genes. Selecting the right treatment for the right patient requires consideration of disease characteristics and prior treatment sequence, as well as patient preferences and comorbidities. The CLL-International Prognostic Index (CLL-IPI) remains the best-validated tool in predicting the time to first therapy among previously untreated patients, which guides selection for early intervention efforts. This review summarizes our current approach to the management of CLL, right from the time of diagnosis through relapsed disease.

## Introduction

The 2022 lymphoid neoplasm classification updates from the World Health Organization and the International Consensus Classification Clinical Advisory Committee agree in defining chronic lymphocytic leukemia (CLL) as a low-grade lymphoproliferative neoplasm with ≥5 × 10^9^/L clonal B-cells in the peripheral circulation that express CD5, CD19, CD20(dim), and CD23 [1, 2]. All cases of CLL are preceded by monoclonal B-cell lymphocytosis (MBL), a pre-malignant condition defined as <5 × 10^9^/L clonal B-cells in the absence of lymphadenopathy, organomegaly, and cytopenias [1]. MBL has been detected in approximately 10–15% of healthy individuals >40 years of age, making it one of the most common premalignant conditions in humans [3, 4]. Aside from the risk of progression from MBL to CLL requiring therapy (estimated at ~1–2% per year for individuals with high-count MBL [defined by a clonal B-cell count between 0.5 and 5 × 10^9^/L]), patients with high- or low-count MBL have an increased risk for infections and lymphoid malignancies [3, 5, 6]. However, individuals with MBL and early stage asymptomatic CLL who do not meet the 2018 International Workshop on CLL [iwCLL] criteria to initiate therapy should be offered close follow-up (“wait and watch”) [7]. This review will focus on our current approach in the management of patients with CLL from the time of diagnosis through the relapsed setting. Although this schema can be used for CLL patients worldwide, variable availability and regulatory approval of many novel agents outside the United States may limit its broader applicability. In this article, corresponding sections of the treatment algorithm Figures are noted in parentheses (Figure number and numbered section within that Figure) preceding the relevant text to facilitate reference between the content areas.

## Management of the patient with previously untreated CLL

### Patients who do not meet the 2018 iwCLL criteria for therapy

**Fig. 1:**
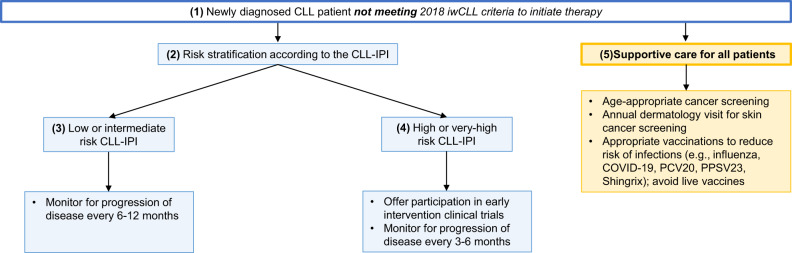
**Approach to the management of patients with newly diagnosed CLL who do not meet the 2018 International Workshop for Chronic Lymphocytic Leukemia (iwCLL) criteria for therapy.** Management is guided by risk-stratification, while including supportive care is necessary for all patients with a CLL diagnosis.

(1.1) The vast majority of CLL patients have early stage asymptomatic disease at diagnosis. Only those patients who meet the 2018 iwCLL criteria [7] for initiation of therapy (Table 1) should be offered treatment (Fig. 1).

**Table 1:** 2018 International Workshop on CLL (iwCLL) guidelines on indications for treatment [7].

Parameter	iwCLL indications for treatment^a^
Lymph nodes	Massive (i.e.,≥ 10 cm), progressive, or symptomatic
Liver and/or spleen size	Massive (i.e.,≥ 6 cm below the left costal margin), progressive, or symptomatic
Constitutional symptoms	Disease-related symptoms^b^
Circulating lymphocyte count	Progressive ≥ 50% over a 2-month period, or lymphocyte doubling time < 6 months^c^
Platelet count	Worsening thrombocytopenia < 100 x 109/L due to progressive marrow failure^d^
Hemoglobin	Worsening anemia < 10 g/dL due to progressive marrow failure^d^
Bone marrow	Progressive marrow failure as per above
Extranodal	Symptomatic or functional extranodal involvement (e.g., skin, kidney, lung, spine)

^a^Autoimmune complications (including autoimmune cytopenias) poorly responsive to corticosteroids or current treatment may represent an additional indication for change in treatment.

^b^Unintentional weight loss ≥ 10% within the previous 6 months; significant fatigue (ECOG performance scale ≥ 2), fevers (38.0 °C) for ≥ 2 weeks without evidence of infection; night sweats for ≥ 1 month without evidence of infection.

^c^Non-CLL factors that may contribute to lymphocytosis (e.g., infections and corticosteroids) should be excluded.

^d^Hemoglobin and platelet count cutoffs require consideration of the rate of decline. In certain patients, counts slightly below these levels may remain stable for an extended period and not require treatment initiation.

#### (1.2) Risk stratification

**Fig. 2:**
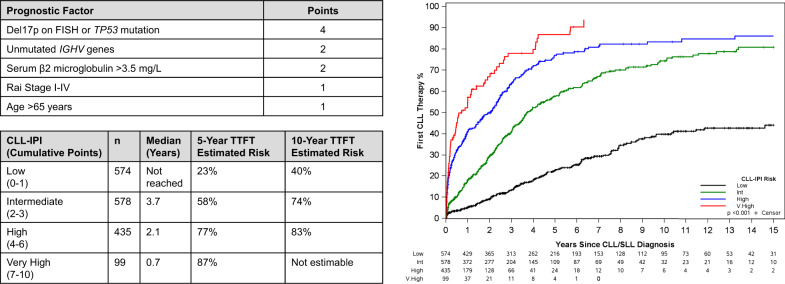
**The CLL-International Prognostic Index (CLL-IPI) and Time to First Therapy (TTFT) among newly diagnosed CLL patients seen at Mayo Clinic, Rochester, MN.** Calculation of the CLL-IPI facilitates prognostic discussions regarding TTFT with newly diagnosed patients with CLL. FISH fluorescence in situ hybridization, IGHV immunoglobulin heavy chain gene, TTFT time to first therapy.

A long list of prognostic markers exists in the CLL literature; a comprehensive review of these is beyond the scope of this management algorithm [8]. In clinical practice, all patients should undergo testing to allow risk stratification according to the CLL International Prognostic Index (CLL-IPI) at the time of diagnosis. The CLL-IPI studied ~28 prognostic variables among ~3400 patients treated on clinical trials across the world and was validated in two independent cohorts of newly diagnosed patients, including from Mayo Clinic and the Scandinavian CLL cohort [9]. Five factors were independently found to be associated with overall survival (OS), including age >65 years, Rai stage I-IV, serum beta-2 microglobulin >3.5 mg/L, unmutated immunoglobulin heavy chain (*IGHV)* genes, and del17p by fluorescence in situ hybridization (FISH) or *TP53* mutation. Four risk groups (low, intermediate, high and very-high risk) with different 5-year OS (93%, 79%, 63%, and 23%, respectively) were identified. Given the rapid adoption of novel agents in the management of CLL, the CLL-IPI can no longer be used to predict OS; however, it is one of the most powerful tools in predicting time to first therapy (TTFT) in patients with previously untreated CLL. Figure 2 shows the time to first CLL therapy in 1,686 patients with newly diagnosed CLL seen at Mayo Clinic in Rochester, MN. The corresponding 5-year risk of needing therapy in the low and intermediate CLL-IPI risk groups was 23% and 58%, respectively, compared to 77% and 87% in the high and very high-risk groups, respectively. Other studies have also confirmed the ability of the CLL-IPI risk score in predicting time to first therapy in previously untreated CLL patients [10–12]. An important caveat to performing prognostic testing in *all* patients with newly diagnosed early stage CLL is that this information may not be necessarily helpful in patients with advanced age, poor performance status, multiple co-morbidities or those with limited life expectancy. Therefore, practicing hematologists/oncologists must exercise their clinical judgment in obtaining these tests in newly diagnosed CLL patients who do not meet indications for therapy.

#### (1.3) Low- or intermediate-risk CLL-IPI

Patients in the low- and intermediate-risk CLL-IPI category (~70% of all newly diagnosed patients, median time to first therapy not reached and 3.7 years, respectively) should be monitored for disease progression every 6-12 months.

#### (1.4) High- or very-high-risk CLL-IPI

Patients in the high- and very high-risk CLL-IPI group (~30% of all newly diagnosed patients, the median time to first therapy 2.1 years and 0.7 years, respectively) should be monitored for disease progression every 3–6 months [9]. Patients with high- and very high-risk CLL may be offered treatment in early intervention clinical trials. The German CLL study group randomized patients with newly diagnosed Binet Stage A CLL patients who had an increased risk of disease progression to either ibrutinib (*n* = 182) or placebo (*n* = 181; CLL 12 trial). After a median follow-up of 31 months, patients who received ibrutinib had a significantly improved event-free survival (event defined as disease progression requiring therapy, time to next CLL treatment or death from any cause) compared to placebo (median not reached vs. 47.8 months, *P* < 0.001), although there was no difference in OS. However, there were significantly more grade 3 adverse events in the ibrutinib arm (including atrial fibrillation, pneumonia, and skin rash) compared to the placebo [13]. Multiple trials are currently ongoing to treat early stage high-risk patients with a variety of different agents [14]. Until mature and long-term results of these trials become available, the current standard for all patients, irrespective of the CLL-IPI risk score at diagnosis, who do not meet 2018 iwCLL criteria for initiation of therapy should still be “watch and wait.” [15]

#### (1.5) Supportive care for all patients

Regardless of the CLL-IPI score, all patients should be counseled for (a) increased risk of infections, with special attention to appropriate vaccinations according to the Centers for Disease Control and Prevention (CDC) guidelines [16]; (b) increased risk of non-hematologic malignancy, and recommendations to follow age-appropriate cancer screening; and (c) increased risk of skin cancers, with yearly full body skin exam by dermatology [17, 18].

### Patients who meet the 2018 iwCLL criteria to start therapy

**Fig. 3:**
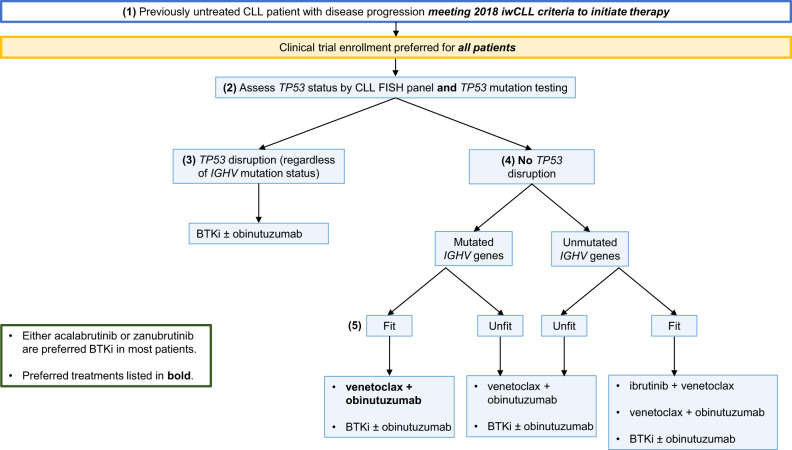
Approach to the management of patients with previously untreated CLL who meet the 2018 International Workshop for Chronic Lymphocytic Leukemia (iwCLL) criteria for therapy. BTKi Bruton tyrosine kinase inhibitors, IGHV immunoglobulin heavy chain gene.

(3.1) All patients who meet the 2018 iwCLL criteria (Table 1) should be offered therapy, regardless of their CLL-IPI risk group assignment (Fig. 3). Given the consistent observations of improved progression-free survival (PFS) and OS with the use of novel agents compared to CIT, we no longer recommend the routine use of FCR (fludarabine, cyclophosphamide, rituximab), BR (bendamustine, rituximab), or chlorambucil in the frontline management of CLL [19–22]. Chemoimmunotherapy may be considered an appropriate treatment option (FCR for <65 years, BR for ≥65 years) who have low-risk cytogenetics, mutated *IGHV* genes, and where novel agents are not easily available. Patient preference and comorbidities play a very important role in choosing therapy, where some patients prefer time-limited venetoclax-based combination therapy compared to oral continuous BTKi. See Fig. 3 for our current approach to the management of previously untreated CLL patients who meet 2018 iwCLL criteria for therapy (outside the context of clinical trials).

#### (3.2) Assess *TP53* status

*TP53* status is one of the most important prognostic and predictive biomarkers in CLL. This should be ascertained using (a) CLL FISH panel to look for evidence of del17p13 *and* (b) Sanger sequencing or next-generation sequencing panel to evaluate for *TP53* mutations, with a cutoff of at least 10%. It is important to obtain both these tests since ~3–5% of patients will harbor a deleterious *TP53* mutation on DNA sequencing in the absence of del17p13 on CLL FISH, and multiple studies have shown these patients have equally poor outcomes [23–27].

#### (3.3) *TP53* Disruption Present

Patients with *TP53* disruption have a short PFS and OS when treated with standard chemoimmunotherapy regimens such as FCR and BR. [28, 29]. In contrast, a phase 2 study of continuous single-agent ibrutinib in previously untreated CLL patients (*n* = 34) with *TP53* disruption showed the 6-year PFS was 61%, and the 6-year OS was 79% [30]. Similar findings were reported from a pooled analysis of four clinical trials where ibrutinib ± rituximab was used in the management of CLL patients with *TP53* disruption (*n* = 89); the 4-year PFS was 79%, and 4-year OS was 88% [31]. The median PFS was 49 months among patients with del17p treated with fixed-duration venetoclax-obinutuzumab [19], suggesting that continuous BTK inhibitor-based treatment in patients with *TP53* disruption may provide superior PFS. There appears to be no consistent benefit with the addition of anti-CD20 antibodies (such as rituximab and obinutuzumab) to monotherapy with BTKi in patients with *TP53* disruption in the frontline management of CLL.

#### (3.4) No *TP53* Disruption

Venetoclax- and BTKi-based treatments are both excellent options for patients without a *TP53* aberration and we favor either over CIT in this setting as well, regardless of *IGHV* mutation status. The following data support our approach.

#### (3.4) No *TP53* Disruption—BTKi ± obinutuzumab treatment

The RESONATE-2 trial compared ibrutinib to chlorambucil in elderly CLL patients (≥65 years; 69% CIRS score>6, del17p patients excluded), and showed that the 7-year PFS was significantly longer with ibrutinib compared to chlorambucil (59% vs. 9%, respectively, *P* < 0.0001). In addition, ibrutinib was also associated with superior OS compared to chlorambucil (median OS not reached vs. 89 months, respectively, crossover allowed from chlorambucil to ibrutinib at disease progression) [32]. The Alliance A041202 study randomized 547 previously untreated CLL patients ≥65 years of age to ibrutinib, ibrutinib-rituximab, or BR (1:1:1 randomization). After a median follow-up of 55 months, patients in the ibrutinib-containing arms had a significantly improved PFS (76% at 4 years) compared to BR (47% at 4 years, *P* < 0.001), although there was no difference in OS [33]. Surprisingly, there was no difference in outcomes between the single agent ibrutinib and ibrutinib-rituximab arms, suggesting that the addition of an anti-CD20 monoclonal antibody did not enhance the activity of ibrutinib. The companion E1912 study in younger CLL patients (<70 years) compared FCR to ibrutinib-rituximab (1:2 randomization) in 529 previously untreated CLL patients without del17p. After a median follow-up of 5.8 years, the 5-year PFS was superior in the ibrutinib-rituximab arm (78%) compared to FCR (51%, *P* < 0.0001), and there was a significant improvement in OS between the two arms (5-year OS: 95% vs. 89%, *P* = 0.02, in favor of ibrutinib-rituximab) [34]. Importantly, patients with mutated *IGHV* genes were also shown to have an improved PFS with the use of ibrutinib-rituximab compared to FCR (5-year PFS: 83% vs. 63%, *P* = 0.001). Finally, the UK FLAIR study also showed the superiority of ibrutinib-rituximab to FCR in 771 previously untreated CLL patients (median PFS not reached for ibrutinib-rituximab versus 67 months for FCR; HR: 0.44; *P* < 0.001) [35].

Other covalent BTK inhibitors, such as acalabrutinib and zanubrutinib—that are more specific for BTK—have been studied in frontline CLL. In the ELEVATE-TN study, acalabrutinib monotherapy was compared to acalabrutinib-obinutuzumab and chlorambucil-obinutuzumab in 535 previously untreated CLL patients (median age = 70 years, 14% patients had del17p). After a median follow-up of 47 months, the estimated PFS at 4 years was 87% with acalabrutinib-obinutuzumab vs. 78% with acalabrutinib monotherapy vs. 25% with chlorambucil-obinutuzumab (*P* < 0.0001 for comparison of acalabrutinib containing regimens to chlorambucil-obinutuzumab) [36]. The SEQUOIA study compared zanubrutinib to BR in 590 previously untreated CLL patients (median age = 70 years, del17p patients excluded); after a median follow-up of 26 months, the estimated 24-month PFS was 85% with zanubrutinib compared to 69% with BR (*P* < 0·0001) [37]. Zanubrutinib does not yet have approval for CLL in the United States but is listed as a “category 1” recommendation on the NCCN Guidelines (Version 1.2023).

There was no difference in outcomes between the single-agent ibrutinib and ibrutinib-rituximab arms in multiple studies; hence, we generally do not use rituximab with BTKi [33, 38]. However, given the PFS benefit noted in ELEVATE-TN, obinutuzumab can be used in combination with acalabrutinib, particularly for patients who do not have *TP53* disruption [36]. Also, we typically include an anti-CD20 monoclonal antibody when a quicker response is required, examples include autoimmune conditions or glomerulonephritis.

#### (3.4) No *TP53* disruption—venetoclax + obinutuzumab treatment

The CLL14 trial compared fixed duration venetoclax-obinutuzumab (venetoclax administered for 12 cycles; each cycle = 28 days) to chlorambucil-obinutuzumab in 432 previously untreated CLL patients (median age = 72 years, median cumulative illness rating scale [CIRS] score = 8, del17p in 8%). After a median follow-up of 52 months, the estimated 4-year PFS was longer with venetoclax-obinutuzumab compared to chlorambucil-obinutuzumab (74% vs. 35%, *P* < 0.0001), although no difference in OS was noted [39]. Initial results from the CLL13 trial (1:1:1:1 randomization to CIT, or one of three venetoclax combinations) showed better undetectable measurable residual disease (uMRD) and PFS with venetoclax + obinutuzumab compared to venetoclax + rituximab, confirming obinutuzumab as the anti-CD20 monoclonal antibody of choice when pairing with venetoclax [40].

#### (3.4) No *TP53* disruption—ibrutinib + venetoclax treatment

A phase 2 study of the combination of ibrutinib and venetoclax (*n* = 80) conducted at the MDACC showed that this combination was able to achieve uMRD in the bone marrow in 56% of patients after 12 cycles of treatment, and the 3-year PFS was 93%. Similar encouraging results were also seen in the CAPTIVATE study—a phase 2 study where all patients received 15 cycles of therapy with ibrutinib and venetoclax (ibrutinib was administered for 3 cycles as monotherapy to reduce the risk of tumor lysis syndrome followed by combination therapy for 12 cycles). In the fixed duration cohort (*n* = 159, where all patients stopped therapy after 15 cycles regardless of MRD status), the best uMRD rates were 60% in the bone marrow, and the 2-year PFS and OS rates were 95% and 98%, respectively [41]. In the MRD cohort (*n* = 154, where patients underwent secondary randomization after 15 cycles based on MRD status), 68% of patients achieved uMRD at the end of treatment [42]. Based on these data, a randomized phase 3 study (GLOW) compared ibrutinib-venetoclax to chlorambucil-obinutuzumab in 211 previously untreated CLL patients (median age = 71 years, del17p excluded). The estimated 30-month PFS rates were 80% for ibrutinib-venetoclax and 36% for chlorambucil-obinutuzumab, with significantly higher uMRD rate with ibrutinib-venetoclax (52% vs. 17%, respectively) [43]. In contrast to the CAPTIVATE study, patients in the GLOW trial were older and experienced more AEs (Grade 3/4 AEs of interest in the GLOW study: diarrhea [10%], atrial fibrillation [6%], infections [15%], and hypertension [7%]). Cross-trial comparisons notwithstanding, we believe the combination of ibrutinib and venetoclax should be preferred only in fit, young individuals with previously untreated CLL, particularly among those with unmutated *IGHV* genes. This combination is not yet approved by the FDA for CLL in the United States but is listed as a “category 2B” recommended regimen on the NCCN Guidelines (Version 1.2023).

#### (3.5) Fitness

Patient fitness can be determined by calculating the CIRS score, as was utilized in the major clinical trials referenced above. A score ≥ 6-8 is generally considered “unfit.” [44] In addition to the options listed in the sections above, single-agent obinutuzumab has efficacy and could be considered in very frail patients with comorbidities that preclude BTKi or venetoclax [45, 46]. Head-to-head prospective studies from the relapsed treatment setting reported more toxicity in patients treated with ibrutinib vs. acalabrutinib or zanubrutinib [47, 48]. Either acalabrutinib or zanubrutinib is a preferred BTKi in most patients.

### Future directions

MRD at the end of CLL therapy (and potentially also as a dynamic assessment) remains a powerful prognostic tool in the novel agent era, particularly with time-limited venetoclax-based approaches [39]. Achieving uMRD with continuous BTKi treatment, in contrast, does not impact PFS [49]. The ongoing phase 3 trials evaluating different combinations of time-limited treatment approaches compared to continuous BTKi ± anti-CD20, in both fixed-duration (EA9161, CLL17) and MRD-directed (A041702, MAJIC) fashion, will shape the treatment landscape over the coming years.

### Patients with relapsed CLL

**Fig. 4:**
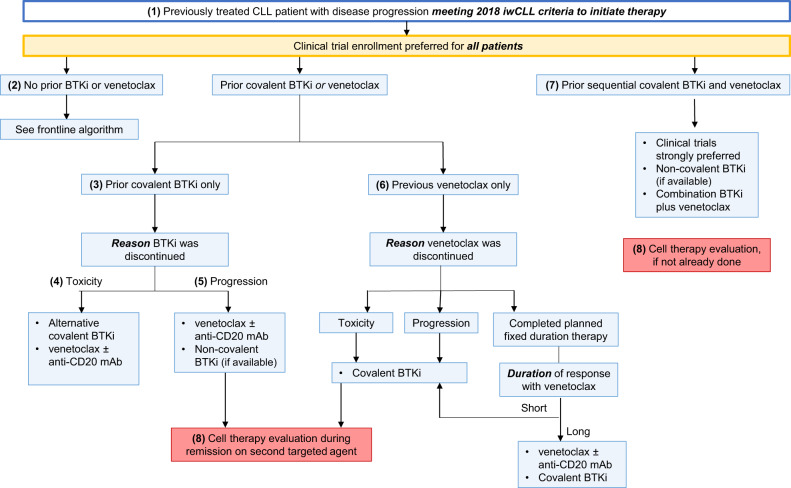
Approach to the management of patients with relapsed CLL who meet the 2018 International Workshop for Chronic Lymphocytic Leukemia (iwCLL) criteria for therapy. BTKi Bruton tyrosine kinase inhibitors, IGHV immunoglobulin heavy chain gene, mAb monoclonal antibody.

(4.1) The progressive disease often does not immediately equate to an indication for starting a treatment or changing the current treatment, until patients meet the 2018 iwCLL criteria for therapy (Table 1; Fig. 4). Patients with relapsed CLL should undergo a comprehensive assessment of their disease status, including bone marrow aspirate and biopsy and typically CT imaging (chest/abdomen/pelvis). A positron emission tomography (PET)/CT scan is preferred if there is suspicion for Richter transformation, and biopsy of lesions with a maximum standardized uptake value (SUVmax) ≥ 5 should be strongly considered (sensitivity 96%, specificity 21%, negative predictive value 86%, among patients experiencing disease progression on BTKi) [50]. *TP53* mutation testing and CLL FISH panel should be repeated, which informs treatment and prognostication similar to the discussion above in the frontline management section. Clonal evolution should also be evaluated via CpG-stimulated karyotyping in all patients, with complex karyotypes (≥3 chromosomal abnormalities) associated with a worse prognosis [51]. Evaluating patients with relapsed CLL also requires the same considerations for medical comorbidities and frailty detailed above in the frontline management section. The added calculus comes with review of prior treatment regimens and outcomes.

#### (4.2) Relapsed CLL in a patient with no prior BTKi or venetoclax treatment

There is no role for CIT in the treatment of patients with relapsed/refractory CLL at the current time. Both BTKi and venetoclax-based treatment options have extensive efficacy and safety data in patients who have previously received CIT and are naïve to novel agents. The shared-decision making process for selecting between BTKi or venetoclax-based treatment options requires similar considerations of medical comorbidities, logistical burden, and patient preferences as outlined in the frontline management section. Discussion of varied toxicity profiles is a critical piece of this discussion, as is the optimal management of adverse events when they arise. These aspects of care are deserving of their own review articles, for which an interested reader has recently published options [52, 53].

#### (4.2) No prior BTKi or venetoclax—BTKi treatment

The phase 3 RESONATE study compared continuous daily ibrutinib to 24 weeks fixed duration ofatumumab in 391 patients with relapsed CLL. In the most recent analysis with up to 74 months follow-up, the median PFS and OS were 44months and 68 months, respectively, with ibrutinib and 8 and 65 months with ofatumumab (crossover to ibrutinib rate of 68%) [54, 55]. ASCEND, a phase 3 study, randomized 398 patients with previously treated CLL (median 2 prior therapies) in a 1:1 fashion to acalabrutinib or the investigator’s choice of idelalisib (continuous) plus rituximab (8 infusions) or BR (6 cycles) [56]. At a median follow-up of ~4 years, the 42-month PFS and OS rates with acalabrutinib were 62% and 78%, respectively, compared to 19% and 65% in the idelalisib plus rituximab or BR arm [57].

Head-to-head prospective trials comparing ibrutinib and second-generation covalent BTKi followed. The first analysis of the open-label phase 3 ELEVATE-RR trial demonstrated similar efficacy (median PFS 38 months in both the acalabrutinib and ibrutinib treatment arms) in a high-risk population (45% with del17p) of 533 patients with relapsed CLL (median 2 prior lines of therapy). However, fewer discontinuations due to adverse events (21% vs. 15%) and less frequent adverse events of interest, including bleeding events (38% vs. 51%), hypertension (9% vs. 23%), atrial fibrillation (9% vs. 16%), and arthralgia (16% vs. 23%) were observed with acalabrutinib compared to ibrutinib [47]. An interim analysis of the first 415 patients treated with ibrutinib or zanubrutinib on the phase 3 ALPINE study similarly showed fewer discontinuations due to toxicities (3% vs. 10%) with zanubrutinib. More mature follow-up is necessary to better understand the efficacy data from this study (including non-pre-specified early analyses) showing higher ORR (78% vs. 63%) PFS rates (12-month rate 95% vs. 84%), and OS rates (12-month 97% vs. 93%) [48]. Therefore, when proceeding with BTKi treatment in the relapsed/refractory setting, we favor acalabrutinib or zanubrutinib given the similar efficacy and less toxicity compared to ibrutinib. Similar considerations for adding anti-CD20 monoclonal antibodies in combination exist here as in the frontline setting.

#### (4.2) No prior BTKi or venetoclax—venetoclax treatment

The phase 3 MURANO study showed superior efficacy with venetoclax (for 2 years) combined with rituximab (6 months) over BR in 389 patients with relapsed CLL (median 1 prior line of therapy; 27% with del17p) [58]. At a median follow-up of 59 months, the median PFS was 53.6 months with venetoclax plus rituximab (vs. 17 months with BR). The 5-year PFS rate was 38% among all patients receiving venetoclax plus rituximab but was lower among those with *TP53* aberrations (27%), unmutated *IGHV* (29%), and genomic complexity (18%, defined by the presence of 3 or more copy number alterations) [59]. Extrapolating from findings in the CLL13 trial, where combination venetoclax plus obinutuzumab appears more effective than venetoclax plus rituximab (3-year PFS 87.7% versus 80.8%, respectively), in practice, we utilize obinutuzumab as our anti-CD20 monoclonal antibody partner of choice with venetoclax when able [40].

Venetoclax monotherapy (given until disease progression or unacceptable toxicity) has also been evaluated, including a phase II study of 158 patients with del17p (median 2 prior lines of therapy) [60]. Extended follow-up demonstrated a median PFS of 28 months and a median OS of 62 months [61]. When used in combination with an anti-CD20 monoclonal antibody, continuation of venetoclax beyond 2 years may be considered in patients with del17p, *TP53* mutation, or those who have detectable MRD at the end of treatment.

#### (4.3) Prior covalent BTKi-exposed/venetoclax-naïve

The reason for prior BTKi discontinuation is the first consideration when determining treatment options for patients previously exposed to a covalent BTKi.

#### (4.4) Covalent BTKi discontinued due to toxicity

BTKi treatment is frequently stopped due to toxicities, such as arthralgia, bleeding, infection, or arrhythmia (50% of discontinuations in routine clinical practice due to toxicity) [62–64]. In this setting, dose reduction may be an option, along with treatment with an alternative covalent BTKi that may extend the collective mileage from this class of treatment. In phase II studies evaluating acalabrutinib after ibrutinib intolerance or zanubrutinib after acalabrutinib or ibrutinib intolerance, the majority of patients (~60%) do not have recurrent toxicity while deriving further clinical benefit [65, 66]. The willingness of a patient and their clinician to consider this option will depend on the specific toxicity and its severity. For example, a patient with myalgias, arthralgias, rash, diarrhea, or even hypertension is a more fitting scenario to consider alternative covalent BTKi than a patient with life-threatening bleeding, recurrent atrial fibrillation, or unexplained cardiac arrest. Again, it is important to consider treating only those patients who meet continued indications for treatment after stopping the covalent BTKi, exemplified in follow-up data from E1912, showing that among patients who discontinued ibrutinib due to toxicity, the median PFS from the time of ibrutinib discontinuation was 25 months [34].

#### (4.5) Covalent BTKi discontinued due to disease progression

Patients with CLL disease progression occurring while receiving a BTKi have the resistant disease and require a different treatment approach. Mechanisms of resistance are similar across covalent BTKi (which share the cysteine 481 binding site), and, therefore, the use of an alternative covalent BTKi will provide no clinical benefit in this setting [67–70].

Venetoclax-based treatment represents the standard of care for patients progressing after BTKi. Prospective data for this approach are mostly limited to a single phase 2 study of venetoclax monotherapy in patients previously treated with ibrutinib (55% with disease progression on ibrutinib). Venetoclax achieved a 65% ORR and 24.7 median PFS in a cohort of 91 heavily pre-treated (median 4 prior lines) patients enriched for del17p (44%) [71]. As the preponderance of data for venetoclax in the BTKi-exposed population come with monotherapy, we consider continuation of venetoclax beyond 2 years in this patient population even when administered with an anti-CD20 monoclonal antibody.

Retrospective data evaluating BTKi-exposed patients have uniformly supported venetoclax-based treatment as an effective option [72, 73]. The largest study to describe survival outcomes with different therapies following the progression of disease on a covalent BTKi reported 29.8 months median OS with venetoclax-based treatment compared to 9.1 months with other approved therapies (i.e., chemoimmunotherapy, PI3K inhibitors, and anti-CD20 monoclonal antibody therapy alone) [74].

A disease flare phenomenon, characterized by rapidly progressive symptoms and adenopathy and rarely histopathologic evidence of Richter transformation, may occur during interruptions in BTKi treatment, but particularly after stopping the covalent BTKi until the next line of therapy is started [63, 75, 76]. We recommend continuing the BTKi during the transition period to next line therapy, particularly through venetoclax ramp-up until the target dose is reached [77].

Non-covalent BTKi, including pirtobrutinib and nemtabrutinib, have shown promising efficacy in BTKi-exposed patients with CLL, including those with or without *C481S* resistance mutations [78, 79]. Furthermore, pirtobrutinib has demonstrated remarkable tolerability with only 1% of patients discontinuing due to a drug-related adverse event. Neither agent is currently available outside of clinical trials as of October 2022, but once approved will represent another option for patients who experience disease progression on a covalent BTKi.

#### (4.6) Prior venetoclax-exposed /BTKi-naïve

Treatment options are guided by the outcome of the prior venetoclax course: stopped due to toxicity, progression while on venetoclax, or progression after fixed-duration therapy.

Covalent BTKi is the best available next option in the setting of an unmanageable toxicity (e.g., neutropenia unsalvageable with growth factor support, refractory diarrhea) or disease progression during venetoclax treatment. A large multicenter study reported a 32-month median PFS with BTKi after venetoclax in BTKi-naïve patients (*n* = 42) [80]. A single-center study of 23 patients with venetoclax-resistant disease showed similar findings with a median PFS of 34 months and 90% ORR [81]. Among the 18 patients who received subsequent BTKi after venetoclax and rituximab in the MURANO study, the ORR was 100% [59].

The decision to restart venetoclax after fixed-duration therapy depends primarily on the duration of the prior response. There is an absence of prospective data to define “long” and “short” remissions. Retreatment with a venetoclax-based regimen should be considered if an interval of ≥1–2 years (“long”) has passed since the completion of venetoclax. Patients with a shorter time from venetoclax completion to disease progression requiring therapy will likely benefit more from a non-venetoclax-based approach.

A phase 2 study evaluating venetoclax retreatment is ongoing (ReVenG NCT04895436), and retrospective data support this approach. An update from the MURANO trial reported a median treatment-free interval of ~24 months among 32 patients who received subsequent venetoclax-based treatment and achieved an ORR of 72% [59]. A multicenter, retrospective study of 46 patients (which included 11 of the aforementioned 32 patients) reported a 76% ORR and median PFS of 25 months with venetoclax-based retreatment after a median of 16 months between completion of the first venetoclax course and the start of the second venetoclax course [82]. *BCL2* mutational testing is commercially available only as part of a larger sequencing panel, and resistance mechanisms to venetoclax have proven more complex than this aberration alone [83]. Currently, assessment for *BCL2* mutations is not part of the decision-making process for repeating venetoclax-based treatment after a prior fixed duration course.

#### (4.7) Prior covalent BTKi and venetoclax

Patients who have sequentially received a covalent BTKi and venetoclax (“double-exposed”) represent an area of unmet need. If prior treatment of either agent was stopped for a reason other than disease progression, then retreatment as per the guidance in the sections above should be considered. In the setting of progression of disease on both agents sequentially (“double-refractory”), no effective treatment options are available. The median OS for patients with CLL progression in this setting has been as short as 8 months [84]. Clinical trial enrollment is particularly critical for these patients. Emerging treatments on the horizon with efficacy in this patient population include the non-covalent BTKi (pirtobrutinib and nemtabrutinib) and chimeric antigen receptor T-cell therapy (CART). Pirtobrutinib achieved a 68% ORR, with responses seen across prior therapy groups. Patients previously treated with a BTKi and BCL2 inhibitor had a median PFS of 18 months with pirtobrutinib [85]. The reported follow-up with nemtabrutinib is comparatively shorter; however, a 58% ORR and median duration of response not evaluable (95% 8.3-not evaluable), including durable responses beyond 20 months in patients with prior BTKi treatment, have been reported [79]. However, non-covalent BTKi and CART are not approved at this time. Small, retrospective studies have suggested revisiting BTKi and venetoclax in combination may provide additional benefit even in double-refractory patients [84, 86, 87]. Achieving even short remissions in this difficult-to-treat population has value in the potential to prolong survival until FDA approval of non-covalent BTKi or facilitate allogeneic hematopoietic stem cell transplantation (HSCT).

#### (4.8) Cell therapy evaluation

CLL remains incurable despite the efficacy of the targeted agents detailed above. Allogeneic HSCT should be considered in patients with appropriate fitness and medical comorbidities during the remission of a second targeted agent, given the poor prognosis and lack of effective options available for double-refractory patients. Allogeneic HSCT achieved 24-month PFS and OS rates of 63% and 81%, respectively, in 65 patients who had received at least one prior targeted agent in the largest multicenter study to date [88].

Although tremendous success has been seen in CLL patients who underwent CART, with some long-lasting remissions, larger studies have failed to routinely achieve these responses, likely due to T-cell dysfunction [89]. In TRANSCEND CLL 004, CD19-directed CART monotherapy achieved an 82% ORR (45% CR), with peripheral blood and bone marrow uMRD rates of 75% and 65%, respectively. The subset of 11 patients with double-refractory disease had similar outcomes (ORR 80%, CR rate 60%, peripheral blood uMRD 78%, bone marrow uMRD 67%) [90]. This study also included a cohort combining lisocabtagene maraleucel with ibrutinib, achieving a 95% ORR (47% CR) and peripheral blood uMRD in 89% (79% in bone marrow) [91]. Complete responses have been achieved with CAR-NK cells in patients with heavily pre-treated, novel agent-exposed disease as well [92]. Optimizing CART and NK cell therapy in patients with CLL remains an area of active research.

### Future directions

Large, cooperative group and international phase III trials that are ongoing or recently completed accrual (EA9161: NCT03701282; A041702: NCT03737981; CLL13: NCT02950051; CLL17: NCT04608318; MAJIC: NCT05057494) include combination venetoclax and BTKi treatment arms in the frontline setting. Data are absent to guide the treatment of patients with CLL relapse on or after time-limited treatment with these targeted agent combination regimens. Clinical trial enrollment, especially for patients with early relapse, is preferred. In the absence of literature to guide us, resuming either agent (BTKi or venetoclax) in a continuous fashion, repeating time-limited venetoclax plus BTKi treatment, or repeating time-limited venetoclax with an anti-CD20 monoclonal antibody (particularly if the anti-CD20 monoclonal antibody was not included in the frontline treatment) represent reasonable options. Non-covalent BTKi or CART therapy would be appealing options if approved. Importantly, no *BTK, PLCG2*, or *BCL2* resistance mutations were identified in patients with progressive disease after fixed duration ibrutinib plus venetoclax on the CAPTIVATE study, suggesting retreatment with either or both of these agents individually is a reasonable strategy [41].

## Special situations

The clinical heterogeneity of CLL includes a multitude of complications requiring special consideration. Management of autoimmune cytopenias, which occur in ~5–10% CLL patients, along with other autoimmune complications, has been covered in recently published reviews [93, 94]. Richter transformation remains a feared complication of CLL in the novel agent era, retaining a poor prognosis. We direct the reader to these recently published papers for the management of Richter transformation of CLL [95, 96].

## Conclusion

Momentous gains in efficacy and tolerability of targeted therapies have continued to shape a dynamic treatment landscape, increasing the ability to individualize treatment to the goals and values of the patient. Pressing questions in the next phase of CLL research include how best to combine novel agents, the sequencing of these treatments, and administering time-limited treatments to achieve deep remissions that allow stopping therapy.

## Data Availability

Since this is a review article, there are no primary data accompanying to share with readers.
