# Correction: Chronic lymphocytic leukemia treatment algorithm 2022

**DOI:** 10.1038/s41408-022-00775-6

**Published:** 2022-12-22

**Authors:** Paul J. Hampel, Sameer A. Parikh

**Affiliations:** Division of Hematology, Mayo Clinic, Rochester, MN USA

**Keywords:** Chronic lymphocytic leukaemia, Disease-free survival

Correction to: *Blood Cancer Journal* 10.1038/s41408-022-00756-9, published online 29 November 2022

In this article, corresponding sections of the treatment algorithm Figures should have been noted in parentheses (Figure number and numbered section within that Figure) preceding the relevant text to facilitate reference between the content areas.

The original article has been corrected.

